# Inflamm-Aging: A New Mechanism Affecting Premature Ovarian Insufficiency

**DOI:** 10.1155/2019/8069898

**Published:** 2019-01-02

**Authors:** Yaoqi Huang, Chuan Hu, Haifeng Ye, Ruichen Luo, Xinxin Fu, Xiaoyan Li, Jian Huang, Weiyun Chen, Yuehui Zheng

**Affiliations:** ^1^Jiangxi Medical College, Nanchang University, Nanchang, China; ^2^Jiangxi Provincial Key Laboratory of Reproductive Physiology and Pathology, Nanchang, China; ^3^The Biobank Center of the Second Affiliated Hospital of Nanchang University, Nanchang 330031, China

## Abstract

The normal function of ovaries, along with the secretion of sex hormones, is among the most important endocrine factors that maintain the female sexual characteristics and promote follicular development and ovulation. Premature ovarian insufficiency (POI) is a common cause in the etiology of female infertility. It is defined as the loss of ovarian function before the age of 40. The characteristics of POI are menstrual disorders, including amenorrhea and delayed menstruation, accompanied by a raised gonadotrophin level and decreased estradiol level. Inflammatory aging is a new concept in the research field of aging. It refers to a chronic and low-degree proinflammatory state which occurs with increasing age. Inflammatory aging is closely associated with multiple diseases, as excessive inflammation can induce the inflammatory lesions in certain organs of the body. In recent years, studies have shown that inflammatory aging plays a significant role in the pathogenesis of POI. This paper begins with the pathogenesis of inflammatory aging and summarizes the relationship between inflammatory aging and premature ovarian insufficiency in a comprehensive way, as well as discussing the new diagnostic and therapeutic methods of POI.

## 1. Introduction

Due to environmental pollution, huge living and working pressures, and other factors, female infertility and premature ovarian insufficiency (POI) have become global issues receiving great attention. POI is a disease defined as the cessation of ovarian function with decreased estrogen levels and elevated gonadotropin levels before the age of 40 [1]. Previous studies have shown that the incidence of POI in women before the age of 40 is approximately 1%, and its incidence in women before 30 is 1‰. Moreover, approximately 10% to 28% of women experienced primary amenorrhea and approximately 4% to 18% of women exhibited secondary amenorrhea [2, 3]. In recent years, studies have shown that inflammatory aging is closely related to POI. Additionally, ovarian biopsy from patients with POI have shown lymphocytic infiltration and other ovarian immune responses [4–8]. Therefore, the role inflammation plays in ovarian function degeneration, as well as the therapeutic methods to delay ovarian aging and improve ovarian function, are currently heavily researched topics in the field of reproduction. The paper will discuss the mechanism of inflammatory aging and its relationship with POI.

## 2. Mechanisms of Inflamm-Aging

**Figure 1:**
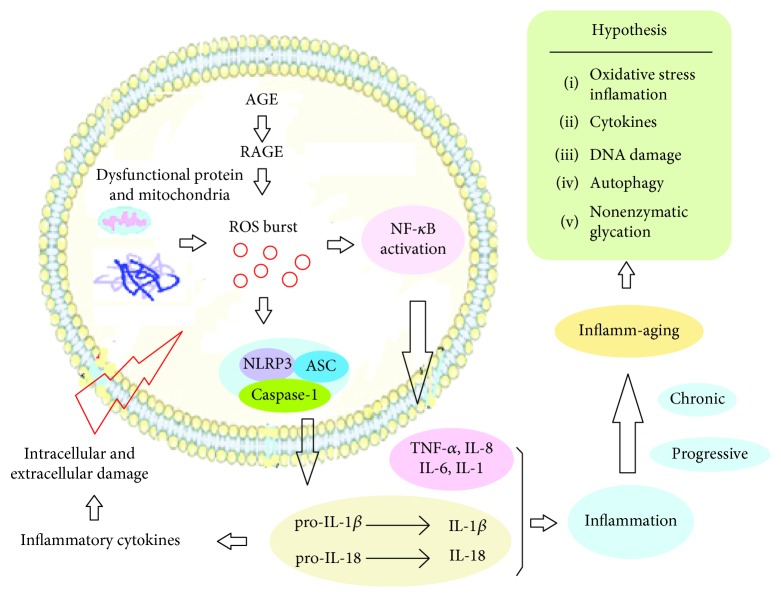
The mechanisms of inflamm-aging. In the process of aging, with the activation of inflammatory factors, the body appears to be in a chronic, progressively elevated proinflammatory state called inflammatory aging. ROS in the body is increased due to several factors. It causes oxidative stress and a series of inflammatory reactions activated by NLPR3 and NF-*κ*B. It is now summarized as follows: oxidative stress inflammation, cytokines, DNA damage, autophagy, and nonenzymatic glycation.

The natural aging of the body is a long and complex biological process, which results from the interaction of intrinsic and extrinsic factors during the body's degeneration period. Aging is characterized by the degeneration of structures; the imbalance of the internal environment; the decline of function; and the loss of adaptability, resilience, and resistance [9]. With the rapid development of science, the study of aging is coming into a new research stage; however, the definite mechanism of aging has not been thoroughly elucidated to date. In recent years, an increasing number of studies have found that inflammation is associated with the occurrence of aging; as in the process of aging, the tissues and organs of the body are accompanied by a chronic, progressive proinflammatory state [10–12]. Inflamm-aging [13] was a term first coined by Franceschi et al. in 2000. At present, the mechanism of inflammatory aging can be illustrated with the hypothesis presented in Figure 1.

### 2.1. Oxidative Stress and Inflammation

Oxidative stress refers to an imbalance between oxidation and antioxidation in vivo, which tends to be oxidative and leads to neutrophil inflammatory infiltration. Oxidative stress is caused by excessive reactive oxygen species (including oxygen free radicals) and plays an important role in the inflammatory aging of the body caused by chronic inflammation [14, 15]. In the 1990s, Sohal and Weindruch [16] noted the defects of the free radical theory and proposed the concept of oxidative stress based on the close relationship between oxidative stress and aging. Ottaviani and Franceschi [17] investigated a series of different species, ranging from invertebrates to humans, and found that immune-stress-inflammation formed a defense network of the body, confirming that stress was one of the causes of inflammatory aging. In addition, antioxidants have been successfully applied to reduce oxidative stress damage and improve the longevity of individuals which also confirm the hypothesis of oxidative stress inflammation [18, 19].

### 2.2. Proinflammatory Cytokines and Aging

Excessive expression of the inflammatory factors will cause a high proinflammatory state in the body, which is an important element in inflammatory aging. A large number of studies have shown that the serum levels of inflammatory factors, such as IL-6, IL-8, TNF-*α*, and PGE2, are significantly higher in aging organs [20–22]. Salvioli et al. [23] examined normal individuals of all ages and patients with diseases related to aging (such as Alzheimer's disease and Type 2 diabetes) and determined that elevated levels of proinflammatory cytokines play an important role in aging.

### 2.3. DNA Damage

The DNA repair mechanism of the normal body can repair damaged DNA, but with increasing age, DNA damage continues to accumulate, eventually leading to cell death. Bonafe et al.'s study proved that the inflammatory aging of the body is due to DNA damage; with stem cells and stromal fibroblasts differentiating, the overexpression of proinflammatory cytokines is triggered, which disintegrates the multishell cytokine network [24].

### 2.4. Autophagy

Professor Christian de Duve proposed the concept of autophagy in the 1960s, and accumulated evidence suggests that autophagy is prevalent in eukaryotic cells and plays a vital role in maintaining cell homeostasis and delaying aging [18, 25, 26]. Salminen et al. [27] found that in the course of aging, the autophagic cleansing capacity declines gradually and dysfunctional protein and mitochondria accumulate, leading to an increased level of reactive oxygen species (ROS) and oxidative stress. ROS activates NOD-like receptor 3 (NLRP3) and causes a series of inflammatory reactions, such as the increased secretion levels of IL-1*β* and IL-18. In turn, these cytokines also accelerate the aging process through inflammation caused by inhibiting autophagy [28].

### 2.5. Glycation

Saccharification is one of the endogenous aging mechanisms. Advanced glycosylation end products (AGEs) are the end products of nonenzymatic glycosylation that accumulate in the body with increasing age [29]. The glycosylation aging theory accepted by many scholars argues that glycosylation will cause the crosslinking damage of proteins, which can convert proteins with normal structures into abnormal aging ones. Studies have shown that the AGE receptor RAGE (receptor for end products of advanced glycation) regulates inflammation, apoptosis, autophagy, senescence, and other important cellular processes by combining and transducing various inflammatory gametes, such as AGEs, S100 calmodulin, and high mobility group box-1 protein (HMGB.1) [30, 31]. Nakashima et al. [32] noted that when comparing the number of glycosylated protein and membrane protein on the erythrocyte membrane which were labelled with fluorescent substances, the fluorescence degree of erythrocytes from older individuals were significantly higher than those from younger individuals. Nonenzymatic glycosylation products can also accelerate body aging by a range of changes, such as the enhancement of lipid peroxidation.

## 3. Inflammatory Aging and Premature Ovarian Insufficiency (POI)

According to the new guidelines of the European Society of Human Reproduction and Embryology, POI is defined as the loss of ovarian activity in a woman before the age of 40 with elevated gonadotrophin and decreased estrogen [3, 33, 34]. The diagnosis of POI requires simultaneous abnormal menstruation and biochemical abnormalities: oligomenorrhea/amenorrhea for at least 4 months and an elevated FSH level > 25 IU/L on two occasions (4 weeks apart) [35]. The patients include women younger than 40 years (which includes Turner Syndrome patients) and women older than 40 years, but older patients must be affected before the age of 40 [1, 36]. The main clinical manifestation is amenorrhea, which is also accompanied by hot flashes, sweating, loss of libido, and other menopausal symptoms. Severe cases can even lead to infertility, greatly affecting the female reproductive function [37].The etiology and pathogenesis of POI are very complex. A mass of exogenous factors are involved, such as surgery, drugs, and the environment, as well as endogenous factors, such as chromosomal linkage defects [38, 39], autoimmune reactions [40, 41], psychological stress [42], genetic predisposition [1, 43], and congenital enzyme deficiencies. With plenty of domestic and foreign research confirming that TNF-*α* and IL-6 may play a role in ovarian function [44, 45], it follows that inflammatory factors may be a vital cause of POI. Thus, this review will discuss the relationship between inflammatory aging and POI from the following perspectives.

### 3.1. Inflammatory Cytokines and Anti-Inflammatory Factors Cause Ovarian Change

In recent years, the role that inflammatory aging plays in ovarian disease has raised great concern, in which follicular rupture is considered as an inflammatory response, and IL-1 and TNF-*α* are the major cytokines involved in this process.

It has been reported that the abnormal performance of xanthogranulomatous inflammation in the female genital tract is a POI [46]. This suggests that inflammatory aging may be one of the causes of POI. Th1 cell- (a type of T helper cells) mediated immune response is often associated with the inflammatory response. The targeting action of inhibin-alpha on the experimental autoimmune ovarian inflammation is initiated by CD4 (+) Th1 T cells, by stimulating B cells to produce inhibin-alpha neutralizing Abs, which directly mediates POI and transfers the disease to the naive receptor [47].

Many molecules involved in the immune response and inflammatory response are regulated by nuclear factor-*κ* gene binding (NF-*κ*B), which includes TNF-*α*, IL-1*β*, IL-2, IL-6, and colony stimulating factor. In addition, zinc finger protein A20 (tumor necrosis factor alpha-induced protein 3), heme oxygenase (HO-1), and many other anti-inflammatory factors are also regulated by NF-*κ*B. NF-*κ*B is a transcription factor that can turn on the genes related to the inflammation and immune responses. Furthermore, this factor can be activated when the proinflammatory cytokines are present. In this process, there are expressions of inflammatory markers, such as IL-6 and IL-8, and the level of anti-inflammatory factor IL-10 mRNA decreased [48]. Scientists detected NF-*κ*B activity in the mouse brain with aging progression, which indicated that the protein has little activity in the hypothalamus of young mice, and with mouse aging, the protein becomes increasingly active.

**Figure 2:**
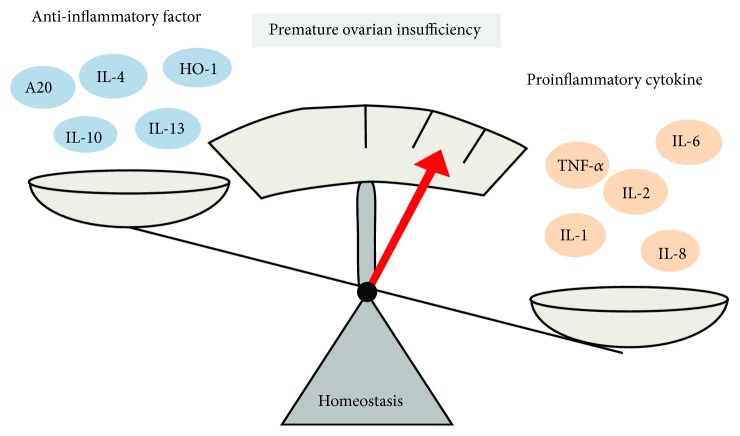
Regulation of proinflammatory cytokines and anti-inflammatory cytokines on premature ovarian insufficiency. Proinflammatory cytokines and anti-inflammatory cytokines maintain a dynamic balance in the normal body. Pathological inflammation in premature ovarian insufficiency is caused by an imbalance of the inflammatory cytokine network.

Thus, elevated levels of inflammatory cytokines and decreased levels of anti-inflammatory cytokines play a critical role in POI (Figure 2).

### 3.2. Changes of Inflammatory Factors in Ovarian Senescence and Reversal

Naz et al. [44] detected the levels of inflammatory factors in patients with POI and normal women using ELISA, and they found that the value of TNF-*α* in patients with POI was lower than that in normal women. Consistent with this result, Wang et al. [49] demonstrated that the level of antizona pellucid antibodies (AzpAb) in POI patients was significantly higher than that in normal controls, and the TNF-*α* and IL-2 levels decreased significantly, whereas INF-*γ* increased significantly. The levels of TNF-*α* and IL-2 in the serum of the POI group were significantly lower than those of the normal group, probably as TNF-*α* was secreted by lymphocytes and granulocytes, while POI patients experience a decreased serum level of the associated inflammatory factors, due to ovarian tissue atrophy and granulocyte reduction. After Sundaresan et al. [50] studied birds, they found that the expression levels of cytokines (IL-1*β*, IL-6, IL-10, and TGF-β2) and chemokines are elevated in POI, which confirmed the relationship between POI and inflammatory aging.

Accordingly, the level of inflammatory factors should decrease after repairing the damaged ovarian function. Recently, studies have shown that synovial mesenchymal stem cells (SMSCs) may play a role in restoring the damaged ovary in the ovarian follicular microenvironment [51]. When SMSCs were used for the treatment of mice, RT-PCR results showed that the expression level of proinflammatory cytokines, such as TNF-*α*, TGF-*β*, IL-8, IL-6, IL-1, and IFN*γ*, is significantly lower than that of the untreated control group of ovaries. When POI occurs, the expression level of certain inflammatory factors increases, while when the damaged ovarian function is repaired, the level of inflammatory factors decreases accordingly, which is sufficient to show that inflammatory aging and POI are closely related.

### 3.3. The Cure of Premature Ovarian Insufficiency by the Treatment of Chronic Inflammation

It is evident that inflammatory aging plays an important part in the pathogenesis of POI, which suggests that we can prevent ovarian insufficiency through anti-inflammation. Said et al. [48] found that resveratrol restores ovarian function by increasing serum levels of the anti-Miller hormone (AMH) and reducing ovarian inflammation, mainly through upregulating the expression of peroxisome proliferator-activated receptors and SIRT1 (sirtuin-1) to inhibit NF-*κ*B-induced inflammatory cytokines. These findings indicate the expression of inflammatory factors such as IL-6 and IL-8. Professor Cai's research team injected mice with GnRH, a hormone produced by the hypothalamus, and mice showed a reduction in signs of aging, which indicated that the inflammatory protein nuclear factor (NF-*κ*B) and the upstream activator IKK-*β* (I*κ*B kinase) inhibited GnRH expression [52]. The antiaging mechanism of GnRH is the hormonal stimulation of the entire cerebral nerve, thereby causing cascading benefits to the entire body. This mechanism may be a new way to regulate aging signals: using anti-inflammatory compounds to treat the brain, which may be able to slow down ovarian aging-related degradation.

He et al. [53] studied the effects and mechanisms of ginsenoside Rg1 acting on the POI induced by D-galactose (D-gal) and found that Rg1 can enhance anti-inflammation and antioxidation. It also reduces the expression of senescence signaling pathway protein, which can reduce damage to the ovary and improve the fertility of POI mice. This study suggests that controlling the development of inflammatory aging may be one of the methods to cure POI. Currently, many inflammatory markers have been identified, such as the plasma tumor necrosis factor, interleukin family, and plasma inflammatory protein which can be used to monitor the ovarian function and cure POI.

However, since there are many other reasons for the occurrence of POI, the pathogenesis is unclear. What anti-inflammatory treatment or other therapeutic methods can make a more remarkable effect? How efficacious is anti-inflammatory treatment on individuals with genetic susceptibility to POI? Can POI be prevented by anti-inflammatory treatment? All these questions have to be answered by further studies.

## 4. Conclusions

POI not only leads to the decline (or even loss) of the female reproductive endocrine function but may also affect women's psychological conditions and the function of other organs. Achievements have been made by experts in the area of inflammation and reproduction, which have improved our understanding of the etiology and pathogenesis of POI, as well as provided useful information for protecting ovarian function, delaying aging, and improving female fertility. However, the regulatory mechanisms of inflammation-mediated aging are still unclear, and further research is needed to address several issues, especially the mechanisms underlying some antiaging substances, like GnRH.

In summary, future research should focus on addressing several problems: (1) How can the specific regulatory mechanism of inflammatory aging be determined? (2) How can the regulation network and key nodes of POI caused by inflammatory aging be determined? (3) How can inflammatory aging be prevented from happening? How can the therapeutic methods be made specific to treat chronic inflammation and prevent ovarian premature aging? (4) Can the genetic susceptibility of POI be treated by anti-inflammatory treatment? (5) How effective are anti-inflammatory agents in the prevention of POI? Are there any adverse effects?

With the development of reproductive medicine, the pathogenesis and mechanism of POI will be better understood. Furthermore, the prevention of POI and restoration of the female reproductive function will become hot topics for researchers both domestic and foreign. In any event, anti-inflammatory aging will become an important strategy in the prevention and treatment of POI.
